# SCN1A Genetic Test for Dravet Syndrome (Severe Myoclonic Epilepsy of Infancy and its Clinical Subtypes) for use in the Diagnosis, Prognosis, Treatment and Management of Dravet Syndrome

**DOI:** 10.1371/currents.eogt.c553b83d745dd79bfb61eaf35e522b0b

**Published:** 2013-04-25

**Authors:** Susan A.R. Stenhouse, Rachael Ellis, Sameer Zuberi

**Affiliations:** Scientific AdvisorUK Genetic Testing Network; West of Scotland Genetic Service, Southern General Hospital, Glasgow, United Kingdom; Royal Hospital for Sick Children

## Abstract

Classic Dravet syndrome is also termed severe myoclonic epilepsy of infancy (SMEI). There are subtle phenotypic variants of Dravet which may have all the features of the syndrome except one, such as without myoclonic seizures, onset in the second year or without generalized spike and wave on EEG. These have been termed borderline variants of SMEI. Rather than ascribing multiple different names to marginally different phenotypes, the term Dravet syndrome is now preferred to describe the group of severe infantile onset epilepsies (OMIM #607208, #182389, #604403) associated with mutations in SCN1A (OMIM *182389). SCN1A-related seizure disorders can be inherited in an autosomal dominant manner but most are due to de novo mutations. SCN1A testing can be done through bi-directional DNA sequencing and multiplex ligation-dependent probe amplification (MLPA) for: 1) individuals with electroclinical phenotype of Dravet Syndrome or clinical sub-types – several seizure types in one individual with onset in infancy, refractory to medication and with generalised spike and wave on EEG, or 2) infants less than 1 year old with 2 or more prolonged hemiclonic febrile seizures in early infancy. Disclaimer: This summary is based on a UK Genetic Testing Network (UKGTN) approved Gene Dossier application.

## Clinical Scenario

Test for epilepsy syndromes associated with mutations in the SCN1A gene including the severe infantile onset epilepsies- typical Dravet syndrome (severe myoclonic epilepsy in infancy) and its borderline subtypes. Dravet syndrome typically presents in the first year of life with prolonged febrile and non-febrile, generalised clonic or hemiclonic epileptic seizures in children with no pre-existing developmental problems. Other seizure types including myoclonic, focal and atypical absence seizures appear between the ages of one and four years. The epilepsy is usually refractory to standard anti-epileptic medication and from the second year of life affected children develop an epileptic encephalopathy resulting in cognitive, behavioural and motor impairment. Seizure types within Dravet syndrome such as status epilepticus may be life threatening and sudden unexpected death in epilepsy can occur. Despite the phenotypic variability within the typical and borderline forms they are now all classified together as Dravet syndrome.

- Referrals made by paediatric neurologists, neurologists, epileptologists, paediatricians, clinical geneticists.
- Sample processed for SCN1A mutation screening.
- Target population includes those with electroclinical phenotype of Dravet Syndrome or clinical sub-types – several seizure types in one individual with onset in infancy, refractory to medication and with generalised spike and wave on EEG or infants less than 1 year with 2 or more prolonged hemiclonic febrile seizures in early infancy.
- The estimated likelihood of detecting an SCN1A mutation in a typical Dravet case is 80-90%.
- This test is for use in the diagnosis, prognosis, treatment and management of Dravet Syndrome.
- OMIM number for disease #607208, #182389, #604403
- Gene – name and description - SCN1A, Sodium channel, neuronal type 1, alpha subunit
- OMIM number for Gene *182389
- Technical Method (s) Bi-directional DNA sequencing
- Multiplex Ligation-dependent Probe Amplification (MLPA)

## Test Description

Peripheral blood sample required.

Diagnostic testing methodologies:(DNA sequencing) Mutation scanning in single direction confirmed in opposite direction and again in an exon specific separate assay. All primers are SNP and BLAST alignment checked. All mutations identified in a previous preliminary project were confirmed using this methodology. This methodology is well established in the laboratory for many disorders.

(MLPA) Use of an MLPA kit designed specifically to pick up deletions and duplications in the SCN1Agene. Exon 21 deletion control identified amongst 10 normal control samples, assay repeated to validate results. This methodology is well established in the laboratory for other disorders.

## Public Health Importance

The estimated incidence of the disease in the UK population is difficult to ascertain as historically this group of epilepsy syndromes have been excluded from epidemiological studies as they have been difficult to diagnose in electro-clinical terms 1 . A recent study based on a UK birth cohort suggested an incidence of at least 1 in 40,000 live births for SCN1A positive Dravet syndrome and 1 in 29.000 for Dravet syndrome as a whole 2 . Dravet syndrome has been misdiagnosed as whooping cough vaccine damage or pertussis encephalopathy^3^ .

Where the mutation is inherited the inheritance pattern is autosomal dominant but most cases are found to be de novo. Familial cases most commonly arise in Genetic Epilepsy with Febrile Seizures plus (GEFS+). The majority of cases are sporadic and the great value of this test is providing an early diagnosis and allowing appropriate treatment. Penetrance is difficult to estimate.

A confirmed diagnosis has implications for treatment stragegies and genetic counseling. It can save many additional costly and invasive investigations. When a diagnosis confirms or supports a clinical suspicion, medication changes may result 4. Anti-epileptic medications such as carbamazepine, lamtrigine and pheytoin can worsen seizures in Dravets syndrome whereas there is evidence from placebo controlled trials that a medication called stirpentol in combination with valproate and clobazam may reduce seizures. Infants with Dravet syndrome suffer from developmental regression and there is good evidence that some of this is due to uncontrolled seizures and abnormal EEG activity (an epileptic encephalopathy). There is clinical justification and evidence from recent research on adults with the syndrome to hope that controlling seizures will reduce the cognitive impairment associated with the syndrome 5.

The clinical features of Dravet Syndrome develop over several years so without the support of molecular genetic testing the diagnosis may not be made until 2-4 years of age. By this time the child may have suffered years of uncontrolled seizures and already have significant cognitive impairment 2 .

## Published Reviews, Recommendations and Guidelines

***Systematic evidence reviews:*** None identified

***Recommendations by independent group: ***UKGTN – Gene Dossier

***Guidelines by professional groups: ***None identified

## Evidence overview

***Analytic Validity*** **: **Test accuracy and reliability in measuring analytes or other entities measured (analytic sensitivity and specificity).

Diagnostic testing methodologies:(DNA sequencing) Mutation scanning in single direction confirmed in opposite direction and again in an exon specific separate assay. All primers are SNP and BLAST alignment checked. All mutations identified in a previous preliminary project were confirmed using this methodology. This methodology is well established in the laboratory for many disorders.

(MLPA) Use of an MLPA kit designed specifically for the SCN1A gene. Exon 21 deletion control identified amongst 10 normal control samples, assay repeated to validate results. This methodology is well established in the laboratory for other disorders.

***Validation***: Clinical Molecular Genetic Society (CMGS) Trainee project: 6/6 mutations were identified in “blind” analysis using conformation sensitive capillary electrophoresis (CSCE). A further panel of 20 patients with varied infantile epileptic encephalopathies, referred from a consultant paediatric neurologist, were screened using CSCE and DNA sequencing. Results were confirmed by bi-directional sequencing.

***Analytical Sensitivity ***is estimated at >98% for bi-directional sequencing, 99.5% when MLPA included based upon our own laboratory test performance experience.

***Clinical Validity*** **: **Test accuracy and reliability in** supporting clinical or public health assessment*.*

In all Dravet syndrome cases the clinical sensitivity is around 80%, rising to 90% in typical Dravet syndrome cases. In our series about 10% of individuals classified as typical Dravet syndrome were not found to have an SCN1A mutation.

The negative predictive value is estimated to be low. Approximately 1/100 of our patients thought to have SMEI/related syndrome (based on clinical, and electro-clinical data) were found not to have an SCN1A mutation. This is most likely to be due to allelic heterogeneity particularly for the related syndromes.

The negative predictive value is estimated to be low. Approximately 1/100 of our patients thought to have SMEI/related syndrome (based on clinical, and electro-clinical data) were found not to have an SCN1A mutation. this is most likely to be due to allelic heterogeneity particularly for the related syndromes.

## Clinical Utility: Net benefit of test in improving health outcomes

When a pathogenic mutation is identified the diagnosis can be made and/or confirmed (i.e. some patients are so young that their epilepsy phenotype has not fully evolved enough for a clinical diagnosis to be made). A confirmed diagnosis has implications for treatment strategies and genetic counselling. It can save many additional costly and invasive investigations. When a genetic diagnosis confirms or supports a clinical suspicion, medication changes may result. Anti-epileptic medications such as carbamazepine, lamotrigine and phenytoin can worsen seizures in Dravet Syndrome whereas there is evidence from placebo controlled trials that a medication called stiripentol in combination with valproate and clobazam may reduce seizures. Infants with Dravet syndrome suffer from developmental regression and there is good evidence that some of this is due to the uncontrolled seizures and abnormal EEG activity (an epileptic encephalopathy). There is clinical justification and evidence from recent research on adults with the syndrome to hope that controlling seizures will reduce the cognitive impairment associated with the syndrome 5 .

We undertook a review of our service using questionnaires to ask carers and physicians their views on genetic testing. 187 carers and 163 physicians responded.

In the carers of the mutation positive group, 87% reported genetic testing helpful, 55% said it led to a change in treatment resulting in fewer seizures. 41% described other changes including improved access to therapies and respite care. In 48%, physicians reported that testing facilitated diagnosis earlier than with clinical and EEG data alone. Molecular testing prevented additional investigations in 67% of cases, altered treatment approach in 69%, helped medication choice in 74% and through medication change improved seizure control in 42%. Carer and physician views correlated significantly with regard to the clinical utility of genetic testing. In addition to confirming a clinical diagnosis, SCN1A genetic testing enabled early diagnosis, influenced treatment-choice and facilitated access to additional therapies in a significant proportion of cases^4^.

UKGTN Testing Criteria: Minimum criteria required for testing to be appropriate as stated in the Gene Dossier.

Electroclinical Phenotype of Dravet Syndrome or clinical subtypes - several seizure types in one individual with onset in infancy, refractory to medication and with generalised spike and wave on EEG OR Infants less than 1 year with 2 or more prolonged hemiclonic febrile seizures in early infancy

## Links

- UKGTN Homepage: http://www.ukgtn.nhs.uk/gtn/Home
- UKGTN Gene Dossier: http://www.ukgtn.nhs.uk/gtn/Information/Services/Gene+Dossiers
- UKGTN Testing criteria: http://www.ukgtn.nhs.uk/gtn/Information/Services/Testing_Criteria;
- GeneReviews: http://www.ukgtn.nhs.uk/gtn/Search+for+a+Test/Search+by+Disease+or+Gene

## Competing interests

The authors have declared that no competing interests exist.
